# Supplementary material for: Splice-Junction-Based Mapping of Alternative Isoforms in the Human Proteome
Source: Cell Rep. Author manuscript; Available in PMC 2020 Jan 15. (PMC6961840; doi:10.1016/j.celrep.2019.11.026)
Supplement: 2 [file NIHMS1546469-supplement-2.zip › DF1/PXD006675/RightCoronaryArtery/RightCoronaryArtery_13_YIF1B_RAQPWPGAAGTPR.pdf]

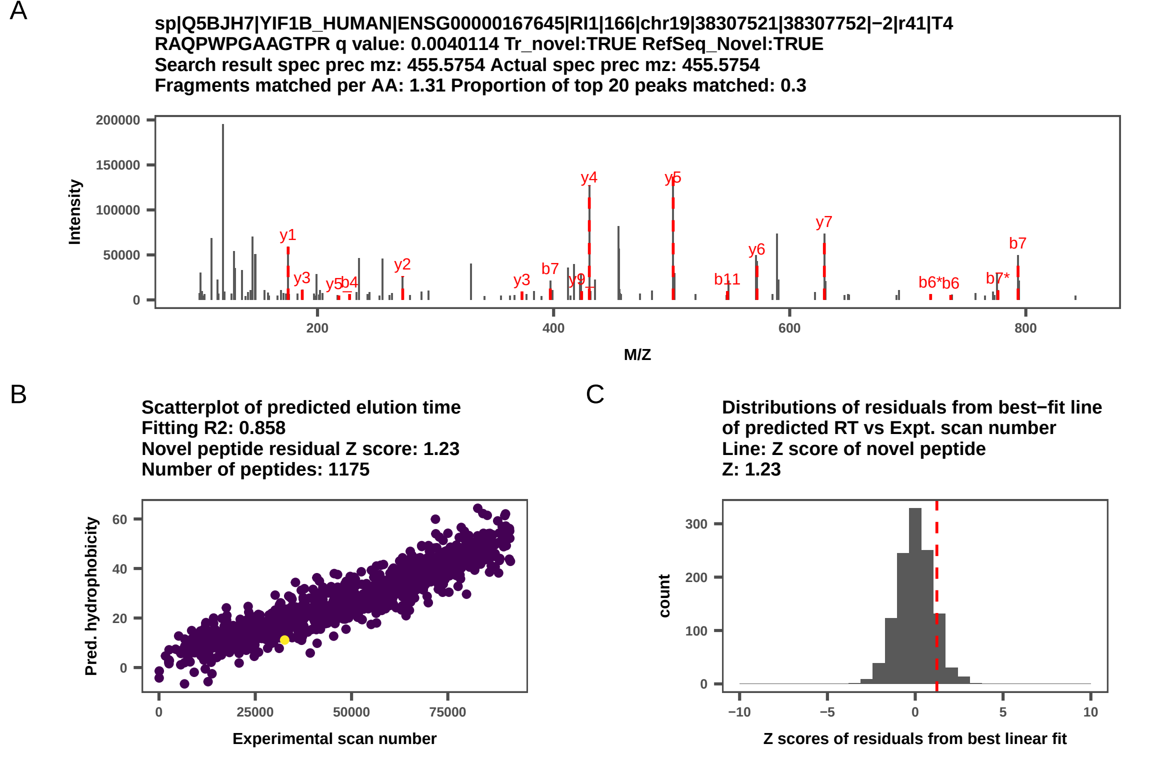
A
sp|Q5BJH7|YIF1B_HUMAN|ENSG00000167645|RI1|166|chr19|38307521|38307752|−2|r41|T4
RAQPWPGAAGTPR q value: 0.0040114 Tr_novel:TRUE RefSeq_Novel:TRUE
Search result spec prec mz: 455.5754 Actual spec prec mz: 455.5754
Fragments matched per AA: 1.31 Proportion of top 20 peaks matched: 0.3
Intensity
200000
150000
100000
50000
0
y1
y3
y5
b4
y2
y3
b7
y9
y4
y5
b11
y6
y7
b6*
b6
b7*
b7
200
400
600
800
M/Z
B
Scatterplot of predicted elution time
Fitting R2: 0.858
Novel peptide residual Z score: 1.23
Number of peptides: 1175
Pred. hydrophobicity
60
40
20
0
0
25000
50000
75000
Experimental scan number
C
Distributions of residuals from best−fit line
of predicted RT vs Expt. scan number
Line: Z score of novel peptide
Z: 1.23
count
300
200
100
0
−10
−5
0
5
10
Z scores of residuals from best linear fit
